# Relationships Between Health and Environmental Information on the Willingness to Pay for Functional Foods: The Case of a New Aloe Vera Based Product

**DOI:** 10.3390/nu11112781

**Published:** 2019-11-15

**Authors:** Castellari Elena, Ricci Elena Claire, Stranieri Stefanella, Marette Stéphan, Sarnataro Martina, Soregaroli Claudio

**Affiliations:** 1 Department of Agri-Food Economics, Università Cattolica del Sacro Cuore, Piacenza, Via Emilia Parmense, 84, 29122 Piacenza, Italy; elena.castellari@unicatt.it (C.E.); m.sarnataro123@gmail.com (S.M.); claudio.soregaroli@unicatt.it (S.C.); 2 Department of Business Administration, Università degli Studi di Verona, Via Cantarane 24, 37129 Verona, Italy; elenaclaire.ricci@univr.it; 3 Department of Environmental Science and Policy, Università degli Studi di Milano, Via Celoria 2, 20133 Milano, Italy; 4 UMR Économie Publique, INRA, Université Paris-Saclay, F-78850 Grignon, France; stephan.marette@agroparistech.fr

**Keywords:** food choices, functional food, health information, environmental information, choice experiment, sustainability

## Abstract

There is an increasing interest in healthy and sustainable product characteristics. Consumers determine their dietary intake and frame production systems with their choices. However, little is known about the relationships between health and environmental information in influencing these choices, especially when considering functional foods. This study assessed the influence of health-related and environmental-friendliness-related product information on the willingness to pay (WTP) for functional foods. To this end, a WTP elicitation experiment was set up using a jam-like fruit compote enriched with *Aloe vera* gel. Participants were provided with different messages related to the health and environmental benefits of *Aloe vera* products, and were also asked to taste the product. Results indicated that providing new information significantly increased the WTP for the enriched compote. This increase was significant for both health and environmentally based benefits, with the health message leading to a higher WTP. Combining health and environmental messages produced an additive effect on WTP which was independent of the sequential order in which the two messages were given. Results contrasted the view that health messages are the main drivers of WTP, and open a broader range of communication in terms of marketing strategies and sustainable policy objectives.

## 1. Introduction

Consumers are increasingly interested in healthy and sustainable product characteristics when buying food [1]. Most consumers have concerns about environmental issues and look for sustainable products with environmentally friendly characteristics which can also positively affect their health [2,3].

In recent decades, the prevalence of food with nutritionally enriched characteristics and functional ingredients aimed at improving human wellbeing has increased considerably [4]. Functional food captures this trend of food demand by offering products that impact positively on human health.

The market for functional food is steadily increasing, even if making an estimate of the market dimension for these products is complicated, mainly because of the lack of a common, internationally recognized definition of such products [5]. In Europe, functional food is defined as “natural or processed foods that contain known or unknown biologically active compounds which, in defined, effective non-toxic amounts, provide a clinically proven and documented health benefit for the prevention, management, or treatment of chronic disease” [6]. Diplock et al. [7] considered a broader definition by considering products to be functional when it has been satisfactorily proven that they positively affect human health and wellbeing beyond nutritional effects. Moreover, Poulsen [8] introduced an even broader definition, identifying four characteristics that could cause a product to be considered functional: (i) the enrichment of food with a substance which is already part of the product; (ii) the substitution of a nutrient with another one; (iii) the adding of a new substance in the product; or (iv) the elimination of a component of the product.

Plant food supplements like aloe-based food products can be considered products with functional characteristics according to the above definitions. Plant food supplements are foodstuffs with a high concentration of botanical preparations that have nutritional or physiological effects, alone or in combination with vitamins, minerals, and other substances which are not plant-based.

Literature to date has explored the market response of functional food mainly by investigating the factors influencing consumer acceptance of such products [9,10,11]. This includes socioeconomic characteristics such as gender [12,13,14], age [15,16], knowledge [17,18,19], and lifestyles [5], as well as cognitive and attitudinal drivers [5,17,20,21,22,23,24] including cultural factors [25], and the role of beliefs [26]. In addition, some quality product characteristics, such as price, convenience, and taste [27] have been found to influence consumer acceptance for functional foods.

A relevant body of literature has also explored the effect of labeled health information on consumer preferences and willingness to use novel foods with functional characteristics. Most of the empirical results have highlighted a positive influence of health claims on the evaluation and choice of functional foods [28,29]. Other studies have concentrated their attention on the information most able to enhance consumer response to health claims for functional foods, highlighting how physiology-related health benefits [30], or health benefits more generally [24,31], are positively impacting motivation to purchase or are evaluated better than nutrition claims or disease-risk claims [17]. At the same time, the negative trade-off between healthy characteristics and taste seems to reduce the acceptance of functional foods over time [27].

To the best of our knowledge, there is currently a gap in the evaluation of other types of information, apart from health-based, on consumer intention to use functional foods. For example, the effect of information about the environmentally sustainable attributes of such products has received only limited attention, even if consumers have been shown to have interest in such characteristics in food products [32,33,34]. Moreover, there is also little evidence on the possible synergic or additive effects of using different types of information on the willingness to pay for products with functional characteristics. Only a recent study conducted by Goetzke et al. [35] has studied the effect of healthy consumer lifestyle on the consumption of organic and functional food. More generally, it appears that the impact of environmental information on food consumption has been much less researched compared to health-related information and the combination of these two types of information has garnered very little attention [36].

Study on the effects of health-related and environmental sustainability information is particularly important as it contributes to the knowledge that could favor a transition toward healthier diets and sustainable food systems [37]. This is a widely recognized goal among scientists, institutions, and the public. Information influences consumer demand, and this demand decides the healthiness of diets and frames production systems [38,39]. There is a growing understanding of the interrelationships between diet, health, and the environment [40]. Actions in this direction are already visible. For example, as highlighted by Hoek et al. [41], although dietary guidelines worldwide are mostly focused on health, some governmental bodies are starting to introduce indications from both environmental and nutritional science to frame new guidelines.

On the basis of the above research gaps, the aims of the present paper were: (i) to assess the influence of health-related and environmental friendliness product information on the willingness to pay for functional foods; (ii) to empirically estimate the presence of possible synergic or additive effects of health and environmental-friendliness-related information on the overall evaluation of functional foods.

In our empirical analysis we referred to a unique, jam-like, aloe-based fruit compote that has not been commercialized. The health characteristics of this product relate to the absence of added sugar and to a high concentration of *Aloe vera*. The gel has a wide array of pharmacological attributes including: anti-viral, anti-bacterial, laxative, protection against radiation, anti-oxidant, anti-inflammation, anticancer, anti-diabetic, anti-allergenic, and immuno-stimulation activities. As far as the food industry is concerned, the potential use of *Aloe vera* gel has mainly focused on the development of functional foods due to its beneficial properties in treating constipation, coughs, diabetes, headaches, arthritis and immune system deficiencies, and digestive effects [42]. The environmentally friendly properties of such products relate to the sustainable cultivation practices of *Aloe vera*, as the plant can grow in arid and marginal areas without the need for chemicals and with a very limited water consumption.

The paper is organized as follows: Section 2 describes the experimental design and the data analysis procedure, Section 3 presents the results, and Section 4 discusses the results in the context of the available literature, and highlights suggestions for future research and study limitations.

## 2. Materials and Methods 

### 2.1. Sample and Products

The experiment was conducted across several sessions in June–July 2017 in Italy. We interviewed 115 respondents in groups of about 15 each, but given the incomplete replies of three participants, the analysis was carried out on a sample of 112. Participants were all aware of the experiment and agreed to be included in the study before participating. Data were treated anonymously and used for research purposes only, with no way of connecting responses to specific individuals.

The experiment focused on two products: (1) a 250 g jar of fruit compote made with *Aloe vera* gel (40%), Sultanina grape (30%), plums (27.5%), and orange peel (2.5%); (2) a 250 g jar of fruit compote with the same composition as the previous one, but with the *Aloe vera* gel replaced with pectin. Pectin was used as it conferred a similar texture to the final product and minimally altered the taste of the product with respect to the other ingredients.

The aloe-based fruit compote was developed by university students taking part in the Ecothrophelia project as an innovative and eco-friendly product called “AloeSpoon”, completely new and original in its category, as at the time the experiment took place, no jam or jam-like products with at least 40% *Aloe vera* gel concentration were available on the market. AloeSpoon is a jam-like fruit compote product that could possibly be assigned to the functional food category thanks to the health benefits conferred by the presence of aloe. The product, as law requires (art. 2, para. 4, Legislative Decree 20 February 2004, no. 50), belongs to the category of “fruit compote” because of its fruit content, which is equal to or higher than 65%. More information about AloeSpoon is provided in Appendix A and Appendix B.

For the purpose of the experiment, what was relevant was the ability to control ceteris paribus for the presence of *Aloe vera* gel. Both products were made in the university laboratory of the Department for Sustainable Food Process of the Università Cattolica del Sacro Cuore, Italy, and were provided to the authors for this experiment. The two fruit compotes were presented using the same type of glass jar, no brand was indicated, and they were visually indistinguishable from each other. Therefore, the only differences perceived by the participants were in the information provided in the experimental design and in the product tasting.

### 2.2. Research Aims and Experimental Design

**Figure 1:**
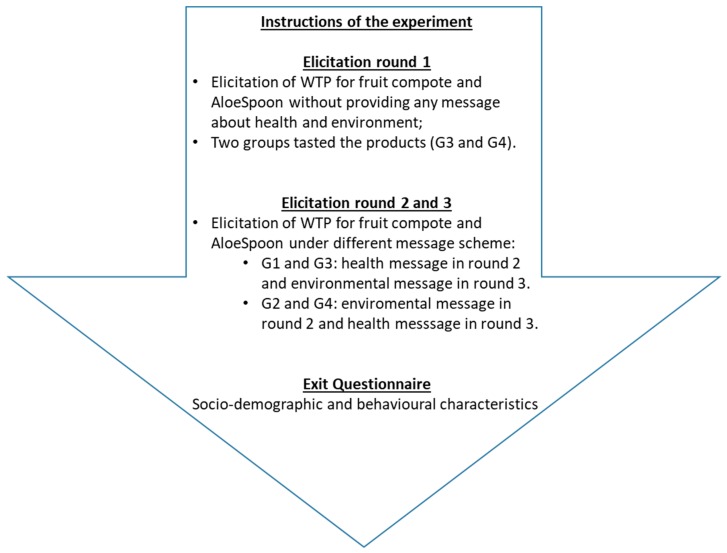
Timeline of the experiment.

This study evaluated the willingness to pay (WTP) for attributes provided by a new (aloe-based) product, AloeSpoon, and the relevance of the information provided. More precisely, the aim of this study was to measure consumers’ WTP for the health and environmental attributes provided by the fruit compote, and to evaluate whether the WTP changed when consumers were informed of the health benefits and of the environmentally friendly characteristics of the product. Moreover, since the product had not been commercialized at the time of the experiment, consumers were not familiar with its taste. This allowed assessment of the possible effect of the *Aloe vera* gel in changing the WTP as a result of product tasting. As explained in Figure 1, product tasting was randomly assigned to two groups (G3 and G4) with different information schemes. The tasting took place at the beginning of the experiment, during the first elicitation round (where no information was provided).

The hypotheses we aimed to test were as follows:

**Hypothesis** **1.** *Consumers assign a higher value to the product enriched with Aloe vera, i.e., they show a higher WTP for such a product compared to its fruit-only counterpart;*

**Hypothesis** **2.** *Providing additional product information on the health-related properties of Aloe vera and/or on the environmental impacts of its production affects the WTP;*

**Hypothesis** **3.** *A health message directed at highlighting the private benefits for consumers is more effective than one about environmental sustainability, which is associated with public benefits, i.e., the health message induces a greater increase in WTP;*

**Hypothesis** **4.** *The two types of information interact and impact the size of their effects;*

**Hypothesis** **5.** *The ordering of the information provided, i.e., whether the health-related or the environmental message is provided first, impacts the evaluation of the product (manifested by different WTP values).*

To this aim, the questionnaire consisted of different rounds of WTP elicitations using a multiple price list (MPL) approach. The MPL approach relies on eliciting WTP asking to the participants their willingness to buy a specific product in an array of ordered prices ranging from a maximum to a minimum. For each price the subject is asked to indicate his willingness to buy indicating “yes”, “no” or “maybe”. The WTP results on the value where the subject switch from “yes” to “no” or “maybe”. In case the subject answer to any price of the array either always “yes” or always “no” or “maybe”, the WTP corresponds either to the maximum or to the minimum of the listed prices. For a further explanation of the MPL and its applications please refer to Andersen et al. [43].

The study was conducted in accordance with the principles of the Declaration of Helsinki. At the beginning of the experiment, initial explanations were read, and participants decided whether they wanted to provide their consent and thus agree to take part in the study. They were informed that all of their replies were anonymous, since they were identified only by a number. Participants were asked to indicate choices as if they were in a supermarket. It was clarified that there were no “good” or “bad” replies, so they were strongly suggested to freely indicate choices reflecting their preferences.

The framing of the experiment in its timeline is illustrated in Figure 1. Following Castellari et al. [44], after providing instructions for the experiment, the first section of WTP elicitation was carried out without any message related to the health or environmental benefits of *Aloe vera*. For this first round, instructions about the experiment were given, with only a few indications describing each product which were basically focused on the composition of the fruit compote.

The health and environmental messages were written after studying articles from the nutrition, agronomic, and environmental fields. The messages were relatively short, because previous works have underlined the benefit of providing a concise message when conveying complex information [45]. The messages provided were the following:

*(a) Health-related message about the benefits of* Aloe vera *consumption*

“Aloe gel has been used for centuries for its healing and therapeutic properties. Frequent use of aloe-based foods brings benefits to health. The gel has anti-viral, anti-bacterial, anti-oxidant, anti-inflammatory, anti-diabetic, anti-allergic, immune- stimulatory, and wetting and wound healing properties, and burn action. It is also used to support the health of the digestive tract. The use of aloe gel in the food industry is mainly focused on the development of functional foods. Thanks to its beneficial properties, it is mainly used in the treatment of constipation, cough, diabetes, migraine, arthritis, and immune system deficiency. Aloe-based products are suitable for consumers of all ages and offer a diverse range of health-related properties”.

*(b) Environmental message about the environmentally friendly characteristics of* Aloe vera

“Aloe vera is an evergreen, xerophytic, greasy plant that has a tissue in the leaves allowing it to store a high water content and to survive in dry regions with reduced rainfall. Thus, it grows and can be cultivated even in arid and/or marginal areas without the need for chemicals, and hence with a highly sustainable agricultural process. It follows, therefore, that the cultivation of aloe has no negative effects on the environment”.

The layout of these messages was precisely controlled by equally varying their order across four different groups of participants (Group1 (G1), Group 2 (G2), Group 3 (G3), and Group 4 (G4). Two groups started with the health-related message preceding the environmental message (G1 and G3), and two other groups started with the environmental message followed by the health-related message (G2 and G4). Furthermore, two of these groups tasted the products during the first round (G3 and G4). Participants were randomly assigned to one of the four groups (G1, G2, G3, G4). (For a clearer picture of the survey structure, refer to Table A2 in Appendix A).

At the end of the third round, participants were asked to fill in a questionnaire aimed at identifying the consumer in terms of sociodemographic characteristics. They were asked to answer general questions concerning their age, gender, job type, family composition, monthly income, purchasing habits, and their previous knowledge about *Aloe vera* and *Aloe-vera-*based products.

### 2.3. Elicitation Mechanism

A multiple price list (payment card) was used to elicit WTP for each product. At the beginning of each round, participants were asked to indicate whether they would be willing to buy the product displayed for prices varying from €2.00 to €18.00 per unit. Research for reference prices was mostly carried out online, since aloe-based products are not usually sold in hypermarkets or large retail chain shops. Price research was also carried out in specialty stores and pharmacies. The multiple price list was characterized by increments of 50 cents between successive prices. For each price, participants were asked to select either “yes”, “no”, or “maybe” regarding their purchase willingness. For each product and each round of choice *R*, with *R* = {1…3}, the WTP was determined by taking the highest price linked to a “yes” choice (with the next highest price on the paper sheet implying a reply “no” or “maybe”). If a participant replied only “no” or “maybe” to each line, the selected WTP was taken as equal to €2.00. If a participant only replied “yes” to each line, the selected WTP was taken as equal to €18.00.

The multiple price list method is a direct survey category which allows consumers to be provided with a product’s description as well as information about its use and benefits. The use of this experimental method has several advantages: First, participants are able to focus on the product attributes rather than just on price. Second, they perceive their role less heavily, since they have some information to rely on. Moreover, participants can better judge the product’s price based on the data provided. Finally, it is relatively easy for subjects to see that truthful revelation is in their best interests: If the subject believes that their responses have no effect on which row is chosen, then the task collapses to a binary choice in which the subject gets what they want if they answer truthfully [46].

Furthermore, the MPL has several attractive characteristics as an elicitation procedure since it is relatively easy to explain to subjects and to implement.

However, some possible disadvantages can be associated with the use of MPL. Firstly, MPL only elicits WTP valuations at intervals instead of “point” estimates [43]. In this experiment, we adopted a €0.5 interval in order to provide an adequate degree of precision for the WTP elicitation.

Secondly, subjects can switch back and forth from row to row, implying potentially inconsistent valuations [43]. This problem was not consistent with the way the consumers were surveyed in this study. Indeed, papers containing different kinds of information were given to consumers separately, in sequential steps.

Lastly, MPL could be susceptible to framing effects, as subjects are drawn to the middle of the ordered table irrespectively of their true values [43]. This can be controlled by changing the price list boundaries; however, we did not do this in the current study [43]. Further analyses by Anderson et al. [46] indicate that multiple price lists perform relatively well, obtaining precise product valuations that are also robust to framing effects.

### 2.4. Sample Description

The final sample analyzed in the experiment was made up of 112 respondents (Table 1). Women represented 60% of the sample, while 40% were men. Of the respondents, 22% of respondents were younger than 30 years of age, 30% were between 30 and 40 years of age, and 47% of the respondents were older than 40 years. A total 36% of the sample had an education up to high school, 32% had obtained a bachelor’s-level degree, and 46% had reached a higher level of education.

These distributions indicate that globally, the sample might have slightly favored consumer segments more interested in the nutritional and environmental aspects of food (for example, for gender and education levels). However, having included such variables in the analyses controlled for possible effects related to sample representitaveness.

### 2.5. Data Analysis

Data analysis included three steps. A first, exploratory step in which descriptive statistics were analyzed was done to provide an initial indication of how consumer preferences changed in the different treatments and rounds.

We then adopted inferential tools to investigate the role of information provision and other variables on consumer willingness to pay. More specifically, we used two random effects Tobit regression models to evaluate whether there was an increased willingness to pay for the compote enriched with *Aloe vera* and whether, and to what extent, this willingness to pay was affected by information provision regarding the health benefits of *Aloe vera*, information provision about the low environmental impacts of *Aloe vera*, or individual socio-demographic characteristics. The choice of the Tobit model was related to the fact that our data was left-censored at €2 and right-censored at €18 because of the experimental design. The random effect was associated to individuals and it was introduced to model possible positive dependencies among answers by the same individuals induced by individual specific preferences toward fruit compotes. The dataset indeed included six answers by each individual, given that answers were pooled for the elicitation round (Round 1 to 3). In detail, we estimated the model as follows:

WTP*_Ri_ = *β*_0_ + *β*_1_*X_R_* + *β*_2_*X_i_* + ν_i_ + *ϵ_Ri_*

*WTP_Ri_* = WTP*_Ri_ if 2 < WTP*_Ri_ < 18 (1)

*WTP_Ri_* = 2 if WTP*_Ri_ ≤ 2

*WTP_Ri_* = 18 if WTP*_Ri_ ≥ 18

where *β*_0_ is the intercept, *β*_1_ is a vector of a parameter associated with the experimental design variables (related to the presence of *Aloe vera*, the information provided, and tasting of the product), *X_R_* is the vector of dummy variables coding the experimental design, *β*_2_ is a vector of a parameter associated with individual characteristics (age, gender, education, income, and occupation), *Xi* is the vector of regressors associated with the individual sociodemographic characteristics, *ν_i_* is the individual specific random effect, and *ϵ_Ri_* is the error term.

More specifically, the two estimated models differed in how we modeled the experimental design. In the first model, X_R_ was a vector of dummy variables indicating the presence of *Aloe vera* (aloe), the provision of a health-related message for the fruit-only/conventional compote (h_conv) or for the *Aloe vera* compote (h_aloe), and the provision of an environmental-friendliness-related message for the fruit-only/conventional compote (en_conv) or for the *Aloe vera* compote (en_aloe). For all of the above dummies, 1 indicated the presence of *Aloe vera* or of the information provision. The same was true for the variable tasting (in both models) which was meant to control for the difference in taste of the two compotes.

Model 2 deepened the investigation into the impact of information provision and, in particular, possible saturation and/or order effects in relation to the health and environmental aspects. In this case, X_R_ was a vector of two variables: treatment and tasting. Tasting (taste variable) was codified as a dummy variable that was assigned a value of 1 when respondents tasted the compote (in groups G3 and G4), and 0 otherwise (in groups G1 and G2). The “treatment” was codified through five dummy variables describing the six treatments: (i) when the WTP was elicited for the fruit-only compote, (ii) for the *Aloe vera* fruit compote with no additional information (Round 1), (iii) for the *Aloe vera* fruit compote with only the health-related information (Round 2 for G1 and G3), (iv) for the *Aloe vera* fruit compote with only the environment-related information (Round 2 for G2 and G4), (v) for the *Aloe vera* fruit compote with the health-related information plus the environmental information—in this order (Round 3 for G1 and G3), and (vi) for the *Aloe vera* fruit compote having received both types of information, but in the opposite order (Round 3 for G2 and G4). This new set of variables allowed us to evaluate ceteris paribus the willingness to pay across the different treatments. As before, we coded 1 when the condition was verified and 0 when it was not.

Finally, we used the delta method to verify via hypothesis tests whether the coefficients that emerged and their differences were statistically different from one other. All analyses were performed using Stata 15.

## 3. Results

Data were first analyzed via descriptive statistics. In particular, we have reported mean, standard deviation, and extreme values for the elicited willingness to pay for the two types of compote in the different experimental conditions (Table 2). What emerged is that the WTP for the aloe compote was always greater that for the conventional fruit-only compote. Moreover, the WTP for the enriched compote increased as more information on the positive properties of *Aloe vera* was given, i.e., from Round 1 to Round 2, and from Round 2 to Round 3 in all cases. However, there seemed to be a greater increase in average WTP when the information regarded health, at least when the first information was provided.

To further investigate the impacts of information and other variables on the willingness to pay for the fruit compotes, we applied a set of regression models. Table 3 reports the results of the first model, while Table 4 presents the results of the second model.

What emerged from the results of Model 1m reported in Table 3, was that the presence of aloe induced a higher willingness to pay for the fruit compote (confirming H.1). Moreover, providing information on the health benefits of *Aloe vera* did not significantly affect the willingness to pay of the conventional fruit compote, but it did significantly increase the willingness to pay for the *Aloe-vera-*enriched compote. Similarly, the provision of the environmental message also did not impact the valuation for the conventional compote, but it increased that for the enriched one (confirming H.2). Tasting the compote with *Aloe vera* did not seem to have a significant effect. Among the individual characteristics, younger respondents seem to value the product less than older ones. Table 3 also indicates that the predicted value of willingness to pay for male respondents was lower compared to that of women. Similar results were found for unemployed consumers. Respondents with higher levels of income were found to have a higher estimated WTP.

The results reported in Table 4 confirmed the strong role of the presence of *Aloe vera* and information provision on the willingness to pay. Indeed, compared to the conventional fruit-only compote, adding *Aloe vera* significantly increased the WTP (confirming H.1). Providing one set of information on either the health benefits or the low environmental impact also significantly increased the WTP for the *Aloe-vera*-enriched compote (confirming H.2). The health message showed a higher coefficient and a greater increase compared to the no information treatment (Table 4). The difference was confirmed statistically by the hypothesis test reported in Table 5 (confirming H.3). 

Moving to Round 3 of the experiment, i.e., when an additional informational message was provided, we found that, again, both types of additional information increased the WTP for the *Aloe-vera*-enriched compote (Table 4). Again, the increase was higher when the message regarded the health benefits compared to the low environmental message (confirming H.3).

Moreover, when we tested the differences between the two health-information-related increases in WTP, we found that they were not significantly different from each other (Table 5). A similar result was found for the environmental messages. Thus, there seemed not to be a diminishing return for the additional information provided nor an amplification of the informational effect; instead, the two effects seem to be additive (confuting H.4).

Furthermore, the final WTP was the same whether the health information was given before or after the environmental information. Indeed, the two coefficients associated with “full” information provision (associated with the variables “aloe_health + env” and “aloe_env + health” in Table 4) were not statistically different from each other (as emerging from Table 5). Thus, the order in which the information was provided did not seem to influence the consumer evaluation of the product elicited as the willingness to pay for it (confuting H.5).

Furthermore, the results reported in Table 4 also indicated that previous knowledge about *Aloe vera* may have had a positive significant effect on the willingness to pay for the *Aloe-vera-*enriched compote.

Table 5 reports the results of the seven hypothesis tests that used the delta method to test whether the regression coefficients β (or some combinations of these) emerging from Model 2 (Table 4) were statistically different from each other.

## 4. Discussion

The presented results lead to several interpretations and implications for practice. First of all, without considering the effect of information, adding a food ingredient such as *Aloe vera* increased the WTP for the fruit compote. This result suggests that the new ingredient changed the reference price thinking of the potential consumer [47]. The absolute value of the price change, which was about €2, also suggested that the product had the potential to move away from the fruit compote category to a “functional” food category. Therefore, it seems that the attribute *Aloe vera* was already perceived as a quality-enhancing attribute, independently of the level of knowledge of the product itself. This introduces a first implication for management: It appears that enhancing an existing product with the *Aloe vera* ingredient might result in a concrete repositioning of the product within its category or even move the product to a different category.

Although *Aloe vera* was perceived to be a quality attribute, the results suggest that there was no full awareness and understanding of its properties. Providing new information significantly increased the WTP for the enriched compote. This increase was significant for both health and environmental-friendliness-based benefits, with the health message leading to a higher WTP as compared to the environmental one. These results confirmed findings highlighting the higher impact of healthiness attributes on motives or WTP of functional food choice [5,24,27] and, more generally, of food categories [48,49]. Low price, taste, and health food attributes are usually seen as better driving food choices compared to environmental ones, because they rely on egoistic motives rather than altruistic ones [40,50,51]. Similarly, in the organic food category, a large body of literature shows that motives for the purchase of organic products are linked mainly to taste and health attributes, and less to environmental aspects [50,52,53]. The importance of egoistic vs. altruistic factors also depends on consumer segments, with the more “conscious” cluster giving additional weight to environmental dimensions [38].

Literature assessing the combined effect of health and environmental messages is scarce. Hoek et al. [36] used a hypothetical choice experiment to investigate the effect of prices and health/environmental logos and information at the point of purchase. Their results emphasized that the effect was very dependent on the consumer segment and on the similarity between standard and alternative products. However, when products were similar, results showed that the combination of health and the environment had a larger effect than when considered independently. The remaining literature has focused more on the potential synergies between healthiness and sustainability attributes, where environmental friendliness attributes could be health-driven and vice versa [40]. For example, Verain et al. [38] highlighted how the perception of healthiness could be increased by a sustainability attribute. They explained it by referring to the so-called halo effect, which is a positive effect on a quality aspect originating from a positive perception of an unrelated attribute of the same product [54,55,56]. In terms of implications for practice and sustainability-enhancing policies, synergies generally lead to the suggestion of preferring egoistic attributes, such as health, to leverage sustainability [38]. Hoek at al. [41] suggested what they called a stealth intervention, where the communication focus remains on health as the main benefit and driver of behavioral change, and environmental friendliness is a side effect.

The presented results are in line with those of Hoek et al. [36]. They showed that a combined effect of health and environmental messages had a larger effect than messages given in insolation. Moreover, findings highlighted how health and environmental messages add up. Both effects were large in magnitude even if, as stated before, the health message had a larger impact. However, the final WTP was independent of the sequential order in which the two messages were given. It could be that both concepts supported each other in a symmetrical way, or that no synergy effect occurred at all. In any case, this result contrasted with the view that health messages should be the main drivers and opened a broader range of communications in terms of marketing strategies and policy objectives. In fact, both concepts could be leveraged and lead to the same outcome, at least in terms of WTP. This result suggests that further investigation is needed on this aspect of functional foods in order to understand the reasons behind this observation and its external validity.

Finally, it has to be noted that tasting was never significant in the presented results. This suggested that the tangible attributes of the augmented product did not significantly affect WTP decisions. The two proposed products were considered very similar, which gave a substantial advantage in identifying the effect of the information component. Controlling for taste was also important as sensorial factors appear to discriminate consumers more than preferences toward health and environment. Consumers need to make a trade-off between decisive factors such as taste and additional attributes such as health [27] or environment [40]. If taste has a neutral effect, it implies that consumers perceive the two products as homogeneous, making it is easier to create a switch from the standard product to the augmented one. Therefore, for a product like the one tested, actions in terms of product pricing could favor a replacement of the standard products with healthier and more environmentally friendly alternatives. Of course, this should consider the cost of the food ingredients, but from a policy perspective this is in line with the results of Hoek et al. [36] showing that a price subsidy for healthy and sustainable food products is effective in shifting consumer choices when products are perceived to be similar. 

The presented research also carried some limitations and areas in need of further investigation. First of all, there is a difference between stated WTP and actual behavior. Participants reported their answers in a controlled environment, which might be substantially different from the contextual situation during a purchase. Information might not be read or noticed in the same way. For this reason, natural field experiments could help to improve the external validity of this type of study. Secondly, functional foods are not a homogeneous category in the eyes of consumers [24], and results should not be generalized to the category. Moreover, the environmental and health-related information was very specific to the combination of the product and *Aloe vera* attribute. Again, external validity could be gained from extending the analysis to other products in the functional domain. Finally, the analysis would benefit from a larger sample that could allow the definition of different clusters of consumers and take cultural effects into account [25,26]. This would allow a better tailoring of marketing and policy communications as, as suggested by Verain et al. [38], health and environmental concepts do not always go hand in hand across consumer segments.

## Figures and Tables

**Table 1:** Sample description.

Variable Name	Variable Definition	Frequency	Percentage
male	1 = if respondent = male; 0 = otherwise	45	40%
		67	60%
younger	1 = if repondent age < 30 years old; 0 = otherwise	25	22%
adult	1 = if respondent was between 30–40 years old; 0 = otherwise	34	30%
older	1 = if respondent was more than 40 years old; 0 = otherwise	53	47%
middle school	1 = if the respondent’s maximum level of education was middle school; 0 = otherwise	3	3%
diploma	1 = if the respondent’s maximum level of education was high school; 0 = otherwise	26	23%
bachelor	1 = if the respondent’s maximum level of education was a bachelor’s degree; 0 = otherwise	32	29%
university	1 = if respondent had a university degree higher than bachelor; 0 = otherwise	51	46%
l_income	1 = if household monthly income was less than €2000, 0 = otherwise	31	28%
m_income	1 = if household monthly income was between €2000–5000; 0 = otherwise	65	58%
h_income	1 = if household monthly income was more than €5000, 0 = otherwise	16	14%
member	1 = if size of family was up to three members; 0 = otherwise	69	62%
		43	38%
unemployed	1 = if respondent was unemployed; 0 = otherwise	15	5%
		321	95%
knowledge	1 = if respondent knew about *Aloe vera*; 0 = otherwise	246	73%
		90	27%

**Table 2:** Descriptive statistics of elicited willingness to pay for the two compotes in the different experimental conditions.

Variable	Experimental Condition	Round	Obs	Mean	Std. Dev.	Min	Max
WTP_conv	No information	1	112	6.0	1.3	2	9
WTP_aloe	No information	1	112	8.1	1.8	4	13
WTP_conv	Health information	2	60	6.2	1.3	3.5	9
WTP_conv	Environmental information	2	52	5.9	1.1	3	8
WTP_aloe	Health information	2	60	9.9	2.1	6	15.5
WTP_aloe	Environmental information	2	52	9.2	1.9	6	15
WTP_conv	Environmental + health information	3	52	6.0	1.3	3	8.5
WTP_conv	Health + environmental information	3	60	6.2	1.4	3.5	9.5
WTP_aloe	Environmental + health information	3	52	10.1	2.2	6	15.5
WTP_aloe	Health + environmental information	3	60	11.0	2.2	7	15.5

*Notes:* In this table we report the descriptive statistics of the willingness to pay (WTP) elicited for the two compotes (conventional fruit-only compote and aloe-enriched compote) in the different treatment conditions. The treatments differed in information provision. The information the subjects were given is reported in the second column. WTPs are measured in euros for a 250 g jar.

**Table 3:** Tobit model with random effect for the WTP for the fruit compote (Model 1).

WTP	*Coef.*		*Std. Err.*	*p-Value*
aloe	1.82	***	0.37	0.000
h_aloe	1.94	***	0.29	0.000
en_aloe	1.80	***	0.41	0.000
h_conv	−0.22		0.29	0.422
en_conv	−0.18		0.41	0.666
taste	−0.27		0.32	0.402
younger	−0.81	**	0.33	0.013
older	−0.06		0.29	0.835
male	−0.63	***	0.18	0.001
l_income	−0.10		0.23	0.639
h_income	1.00	***	0.18	0.000
diploma	−0.04		0.32	0.894
bachelor	0.19		0.35	0.582
university	0.32		0.37	0.379
unemployed	−0.69	**	0.30	0.024
knowledge	0.57		0.35	0.108
_cons	6.37		0.42	0.000
*Obs.*	672			
*Prob > chi2*	0.000			
*LR test of sigma_u = 0*	0.000			

Significance levels: *** *p* ≤ 0.01; ** 0.01 < *p* ≤ 0.05. Notes: Reference categories: age between 30 and 40; income level between €2000–5000; lowest education level (middle school). This table reports the estimated coefficients, standard errors, and related *p*-values associated with all the independent variables included in Model 1. The model applied was a random effect Tobit model censored between €2 and €18 investigating the willingness to pay (WTP) for the fruit compote depending on its attributes (e.g., with/without *Aloe vera*; with/without the health and/or environmental information).

**Table 4:** Tobit model with random effect for the WTP for the fruit compote (Model 2).

WTP	*Coef.*		*Std. Err.*	*p-Value*
Treatment				
aloe_no info	1.78	***	0.36	0.000
aloe_health info	3.40	***	0.37	0.000
aloe_env info	2.87	***	0.37	0.000
aloe_health+env	4.47	***	0.37	0.000
aloe_env+health	4.54	***	0.37	0.000
taste	−0.25		0.31	0.427
Control variables				
younger	0.03		0.23	0.876
older	0.06		0.19	0.761
male	−0.15		0.14	0.288
l_income	−0.05		0.18	0.775
h_income	0.08		0.15	0.576
diploma	−0.05		0.25	0.852
bachelor	−0.14		0.27	0.592
university	0.06		0.28	0.816
unemployed	−0.05		0.23	0.826
knowledge	0.60	*	0.35	0.084
_cons	6.10		0.31	0.000
*Obs.*	672			
*Prob > chi2*	0.000			
*LR test of sigma_u = 0*	0.000			

Significance levels: *** *p* ≤ 0.01; * 0.05 < *p* < 0.1. Notes: This table reports the estimated coefficients, standard errors, and related *p*-values associated with all the independent variables included in Model 2. The model applied was a random effect Tobit model censored between €2 and €18 investigating the willingness to pay (WTP) for the fruit compote compared to the *Aloe-vera*-enriched one in the different treatment conditions.

**Table 5:** Hypothesis testing on the statistical difference among Model 2 regression coefficients β.

*Test*	*Coef.*		*Std. Err.*	*p-Value*
βAloe_Health Info - βAloe_No Info (Test H.2)	1.63	***	0.12	0.000
βAloe_env info - βAloe_no info (test H.2)	1.10	***	0.13	0.000
(βAloe_health info - βAloe_no info) - (βAloe_env info - βAloe_no info) (test H.3)	0.53	***	0.16	0.001
(βAloe_env + health - βAloe_env info) - (βAloe_health + env - βAloe_health info)	0.50	***	0.18	0.006
(βAloe_env info - βAloe_no info) - (βAloe_health + env - βAloe_health info) (test H.4)	−0.06		0.17	0.701
(βAloe_health info - βAloe_no info) - (βAloe_env + health - βAloe_env info) (test H.4)	−0.04		0.17	0.804
(βAloe_health + env - βAloe_env + health) (test H.5)	0.03		0.15	0.859

Significance levels: *** *p* ≤ 0.01. *Notes:* Tests were performed using the delta method on the regression coefficients β derived from Model 2. The model is reported in Equation (1) (Section 2.5), while the estimated regression coefficients are shown in Table 4.

**Figure A1:**
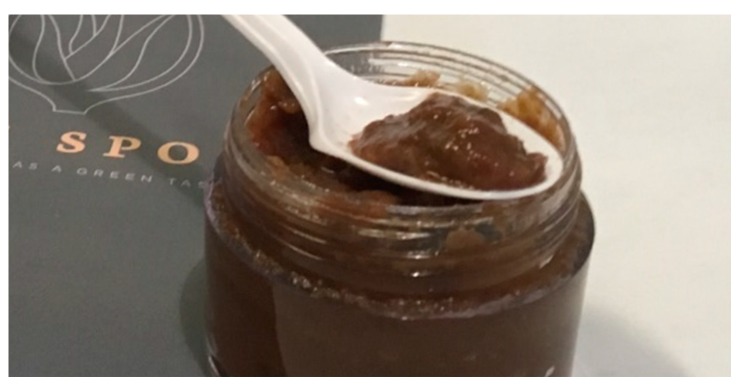
Fruit Compote Visual Appearance. Note: (1) visual appearance did not change between the fruit compotes (Aloe vera gel vs. pectin); (2) no specific labels were used and the jars were the same for the two products.

## Appendix A. Additional Information on the Experimental Settings

**Table A1:** Average nutritional contents of the fruit compotes used in the experiments.

Average Nutritional Values	Per 100 g (% RI)	Per serving (20 g), (% RI)
Calories	795 kJ/190 kcal (9.5%)	159 kJ/38 kcal (1.9%)
Total fat	0.83 g (1%)	0.16 g (<1%)
Saturated fat	0 g (0%)	0 g (0%)
Total carbohydrate	39.7 g (15%)	7.94 g (3%)
Sugars	36.97 g (41%)	7.4 g (8%)
Dietary fibre	4 g	0.8 g
Protein	3.87 g (8%)	0.77 g (1%)
Sodium	1.01 mg (<1%)	0.202 mg (<1%)

Source: elaborations of the nutritional lab of Department for Sustainable Food Process, Università Cattolica del Sacro Cuore, Italy. Note: (1) this information was available during the experiment but not provided to participants unless explicitly requested; (2) if asked, to better control for the effect of information, the nutritional contents of the two fruit compotes were generically stated to be “similar”; (3) only a few participants asked this information and only at the end of the experiment.

**Table A2:** Experimental Conditions.

				Treatment Name	
		G1	G2	G3	G4
Information provided	Round 1	No info	No info	No info	No info
	Round 2	Health	Environment	Health	Environment
	Round 3	Environment	Health	Environment	Health
Taste		No	No	Yes	Yes
Participants (n)		30	30	30	22

## Appendix B. Fruit Compote Composition and Production

AloeSpoon is made of *Aloe vera* gel (40%), Sultanina grape (30%), plums (27.5%), and orange peel (2.5%). The product only contains the sugars that are naturally present in the fruits used in the production process. Aloe gel contains between 97.5% and 98.5% water, followed by beta-polysaccharides; therefore, the caloric value of *Aloe vera* gel is very low and the consumption of one serving (about 200 mL) of gel contributes less than 5 kcal. The biggest intake of simple sugars is provided by Sultanina grape: it is a dehydrated food, so it has a low water content and is a real nutritional and caloric concentrate, in particular because of its high fructose content. Finally, the presence of orange peels allowed several goals to be achieved including a better taste, given that *Aloe vera* tastes naturally bitter.

The production process includes fruit mixing and concentration under heating until the desired level of Brix, measured by refractometer, is reached. *Aloe barbadensis* Mill. gel is then added. The final compote is dosed in the jar, pasteurized, and cooled to ensure the creation of vacuum. The product can be stocked at room temperature.
